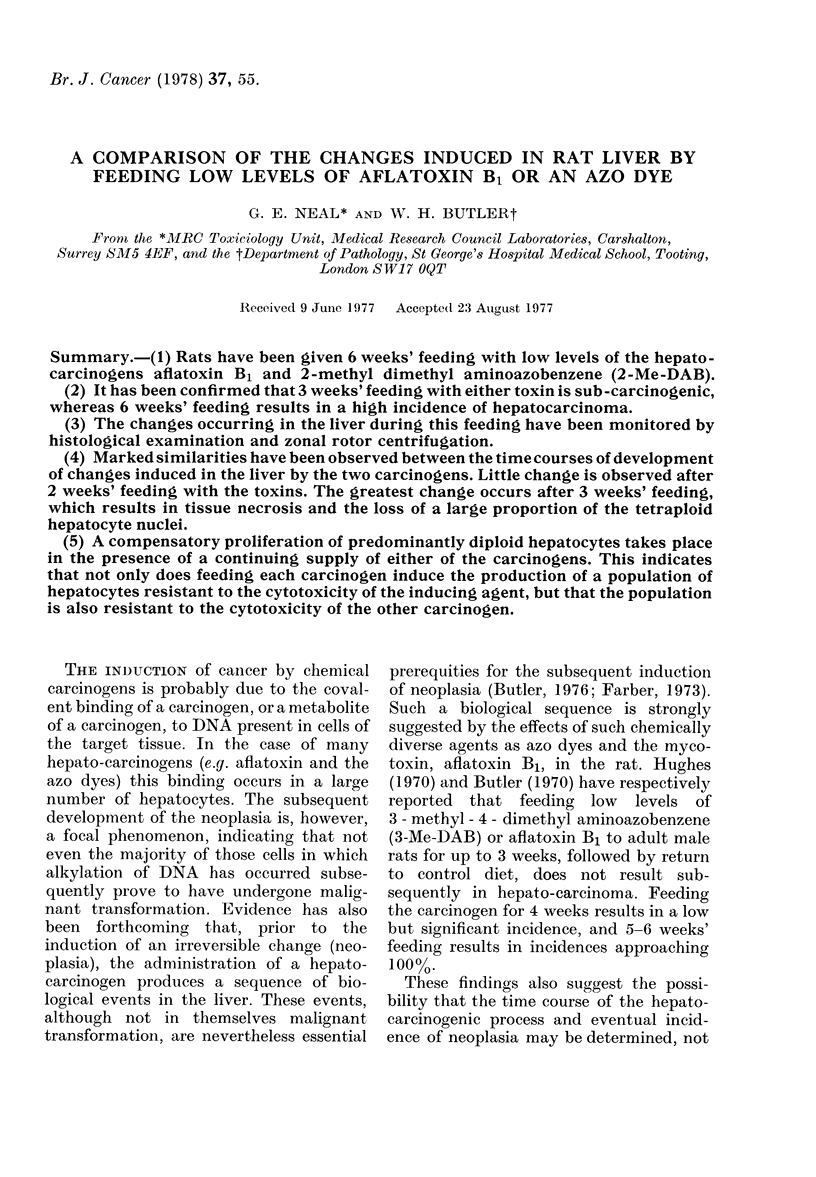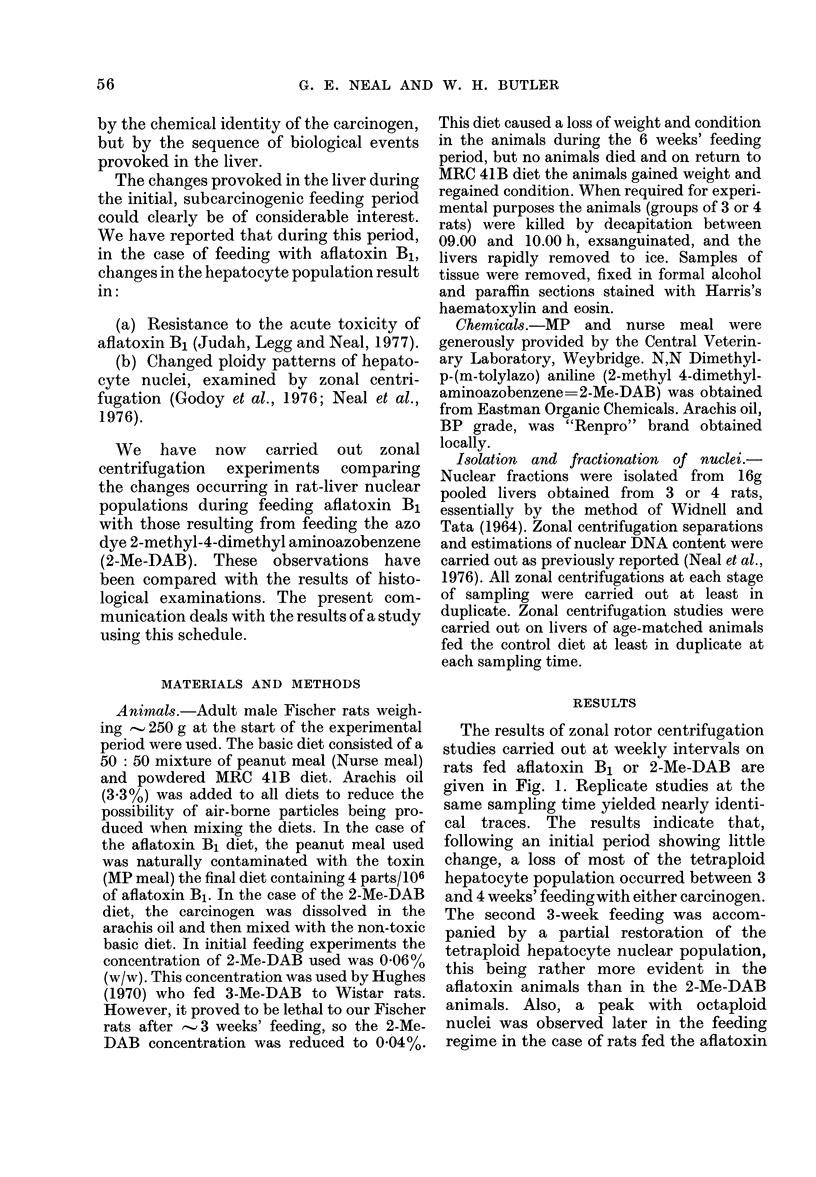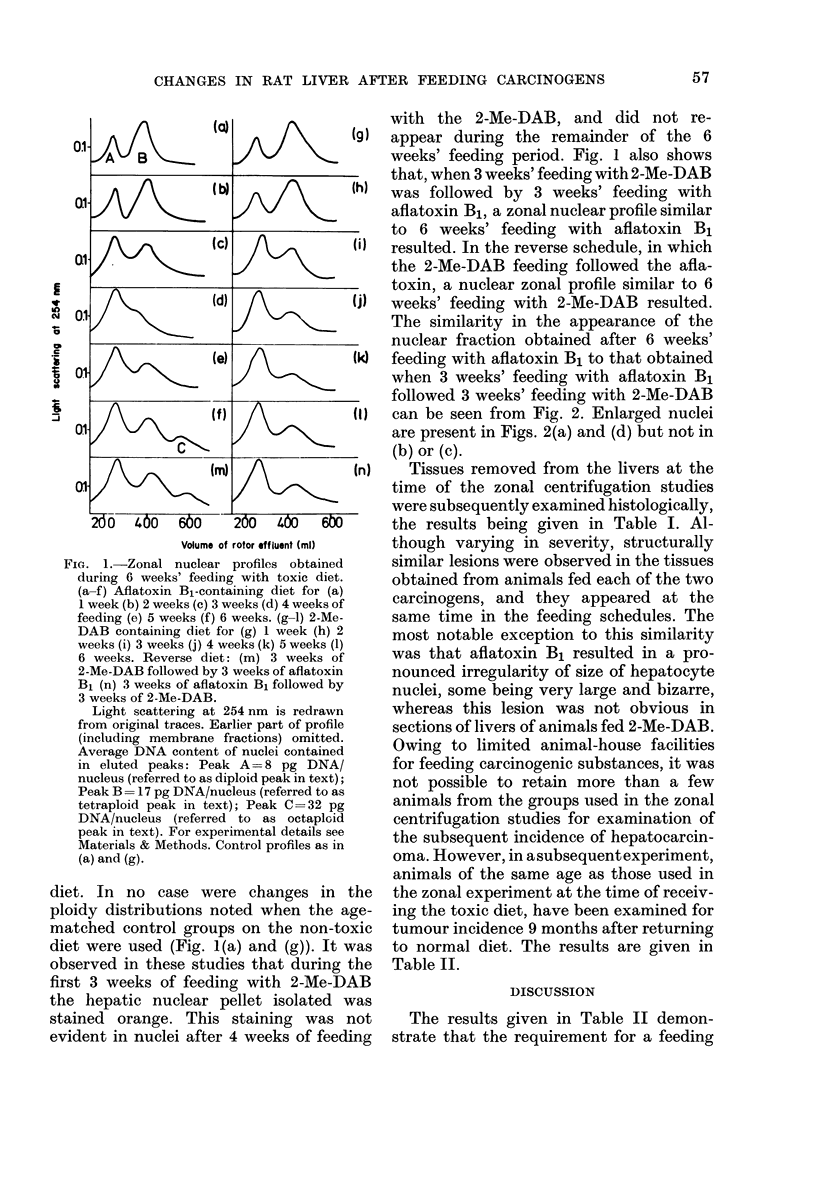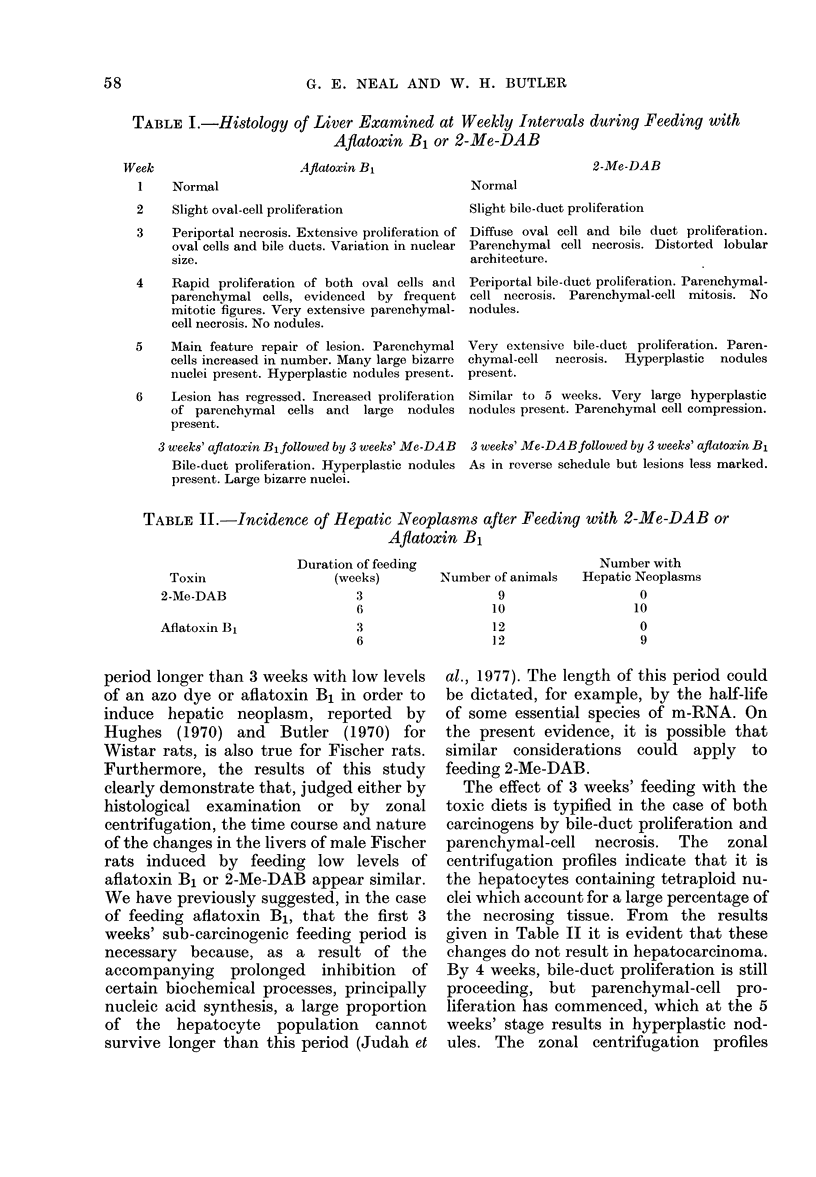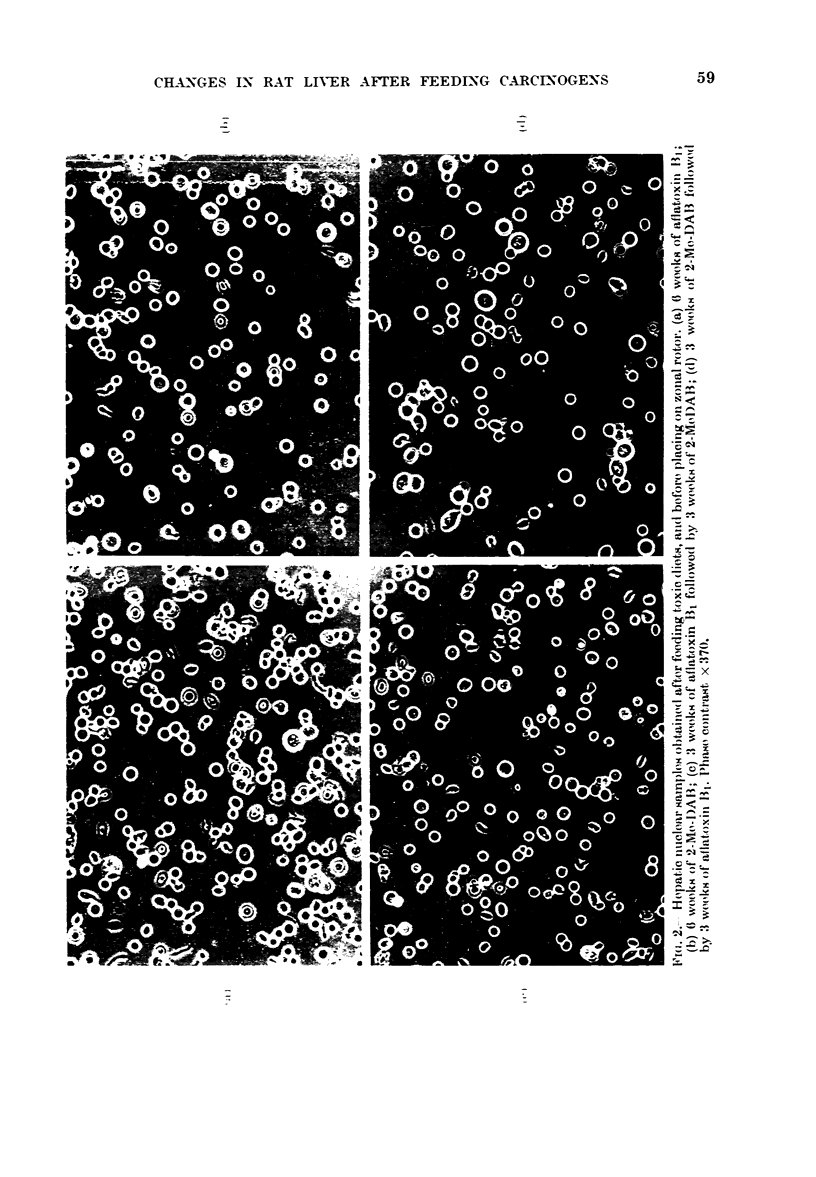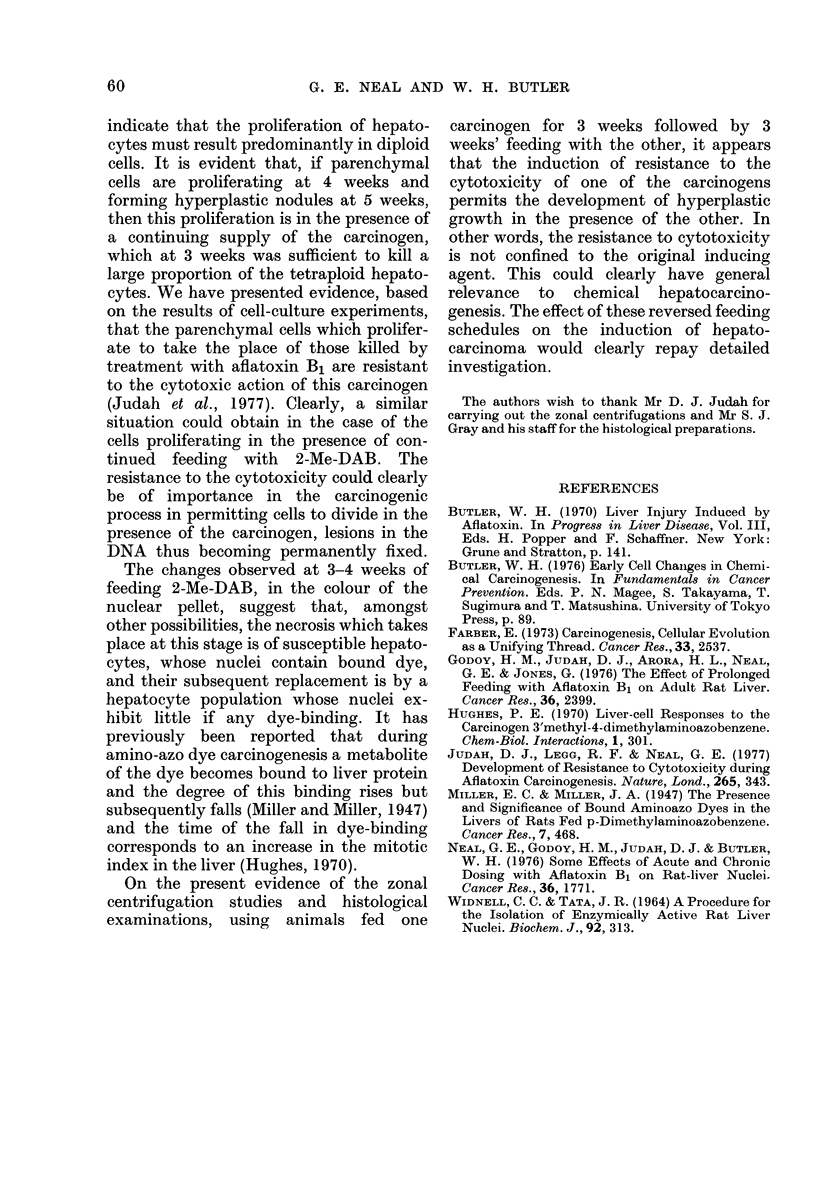# A comparison of the changes induced in rat liver by feeding low levels of aflatoxin B1 or an azo dye.

**DOI:** 10.1038/bjc.1978.8

**Published:** 1978-01

**Authors:** G. E. Neal, W. H. Butler

## Abstract

**Images:**


*Br. J. Cancer* (1978) **37**, 55.

### A COMPARISON OF THE CHANGES INDUCED IN RAT LIVER BY FEEDING LOW LEVELS OF AFLATOXIN $B_1$ OR AN AZO DYE

G. E. NEAL* AND W. H. BUTLER†

*From the *MRC Toxiciology Unit, Medical Research Council Laboratories, Carshalton, Surrey SM5 4EF, and the †Department of Pathology, St George's Hospital Medical School, Tooting, London SW17 0QT*

Received 9 June 1977 Accepted 23 August 1977

**Summary.—(1) Rats have been given 6 weeks' feeding with low levels of the hepatocarcinogens aflatoxin $B_1$ and 2-methyl dimethyl aminoazobenzene (2-Me-DAB).**

**(2) It has been confirmed that 3 weeks' feeding with either toxin is sub-carcinogenic, whereas 6 weeks' feeding results in a high incidence of hepatocarcinoma.**

**(3) The changes occurring in the liver during this feeding have been monitored by histological examination and zonal rotor centrifugation.**

**(4) Marked similarities have been observed between the time courses of development of changes induced in the liver by the two carcinogens. Little change is observed after 2 weeks' feeding with the toxins. The greatest change occurs after 3 weeks' feeding, which results in tissue necrosis and the loss of a large proportion of the tetraploid hepatocyte nuclei.**

**(5) A compensatory proliferation of predominantly diploid hepatocytes takes place in the presence of a continuing supply of either of the carcinogens. This indicates that not only does feeding each carcinogen induce the production of a population of hepatocytes resistant to the cytotoxicity of the inducing agent, but that the population is also resistant to the cytotoxicity of the other carcinogen.**

THE INDUCTION of cancer by chemical carcinogens is probably due to the covalent binding of a carcinogen, or a metabolite of a carcinogen, to DNA present in cells of the target tissue. In the case of many hepato-carcinogens (*e.g.* aflatoxin and the azo dyes) this binding occurs in a large number of hepatocytes. The subsequent development of the neoplasia is, however, a focal phenomenon, indicating that not even the majority of those cells in which alkylation of DNA has occurred subsequently prove to have undergone malignant transformation. Evidence has also been forthcoming that, prior to the induction of an irreversible change (neoplasia), the administration of a hepatocarcinogen produces a sequence of biological events in the liver. These events, although not in themselves malignant transformation, are nevertheless essential prerequities for the subsequent induction of neoplasia (Butler, 1976; Farber, 1973). Such a biological sequence is strongly suggested by the effects of such chemically diverse agents as azo dyes and the mycotoxin, aflatoxin $B_1$, in the rat. Hughes (1970) and Butler (1970) have respectively reported that feeding low levels of 3 - methyl - 4 - dimethyl aminoazobenzene (3-Me-DAB) or aflatoxin $B_1$ to adult male rats for up to 3 weeks, followed by return to control diet, does not result subsequently in hepato-carcinoma. Feeding the carcinogen for 4 weeks results in a low but significant incidence, and 5–6 weeks' feeding results in incidences approaching 100%.

These findings also suggest the possibility that the time course of the hepatocarcinogenic process and eventual incidence of neoplasia may be determined, not by the chemical identity of the carcinogen, but by the sequence of biological events provoked in the liver.

The changes provoked in the liver during the initial, subcarcinogenic feeding period could clearly be of considerable interest. We have reported that during this period, in the case of feeding with aflatoxin $B_1$, changes in the hepatocyte population result in:

(a) Resistance to the acute toxicity of aflatoxin $B_1$ (Judah, Legg and Neal, 1977).

(b) Changed ploidy patterns of hepatocyte nuclei, examined by zonal centrifugation (Godoy *et al.*, 1976; Neal *et al.*, 1976).

We have now carried out zonal centrifugation experiments comparing the changes occurring in rat-liver nuclear populations during feeding aflatoxin $B_1$ with those resulting from feeding the azo dye 2-methyl-4-dimethyl aminoazobenzene (2-Me-DAB). These observations have been compared with the results of histological examinations. The present communication deals with the results of a study using this schedule.

### MATERIALS AND METHODS

*Animals.*—Adult male Fischer rats weighing $\sim$250 g at the start of the experimental period were used. The basic diet consisted of a 50 : 50 mixture of peanut meal (Nurse meal) and powdered MRC 41B diet. Arachis oil (3·3%) was added to all diets to reduce the possibility of air-borne particles being produced when mixing the diets. In the case of the aflatoxin $B_1$ diet, the peanut meal used was naturally contaminated with the toxin (MP meal) the final diet containing 4 parts/$10^6$ of aflatoxin $B_1$. In the case of the 2-Me-DAB diet, the carcinogen was dissolved in the arachis oil and then mixed with the non-toxic basic diet. In initial feeding experiments the concentration of 2-Me-DAB used was 0·06% (w/w). This concentration was used by Hughes (1970) who fed 3-Me-DAB to Wistar rats. However, it proved to be lethal to our Fischer rats after $\sim$3 weeks' feeding, so the 2-Me-DAB concentration was reduced to 0·04%. This diet caused a loss of weight and condition in the animals during the 6 weeks' feeding period, but no animals died and on return to MRC 41B diet the animals gained weight and regained condition. When required for experimental purposes the animals (groups of 3 or 4 rats) were killed by decapitation between 09.00 and 10.00 h, exsanguinated, and the livers rapidly removed to ice. Samples of tissue were removed, fixed in formal alcohol and paraffin sections stained with Harris's haematoxylin and eosin.

*Chemicals.*—MP and nurse meal were generously provided by the Central Veterinary Laboratory, Weybridge. N,N Dimethyl-p-(m-tolylazo) aniline (2-methyl 4-dimethyl-aminoazobenzene=2-Me-DAB) was obtained from Eastman Organic Chemicals. Arachis oil, BP grade, was "Renpro" brand obtained locally.

*Isolation and fractionation of nuclei.*—Nuclear fractions were isolated from 16g pooled livers obtained from 3 or 4 rats, essentially by the method of Widnell and Tata (1964). Zonal centrifugation separations and estimations of nuclear DNA content were carried out as previously reported (Neal *et al.*, 1976). All zonal centrifugations at each stage of sampling were carried out at least in duplicate. Zonal centrifugation studies were carried out on livers of age-matched animals fed the control diet at least in duplicate at each sampling time.

### RESULTS

The results of zonal rotor centrifugation studies carried out at weekly intervals on rats fed aflatoxin $B_1$ or 2-Me-DAB are given in Fig. 1. Replicate studies at the same sampling time yielded nearly identical traces. The results indicate that, following an initial period showing little change, a loss of most of the tetraploid hepatocyte population occurred between 3 and 4 weeks' feeding with either carcinogen. The second 3-week feeding was accompanied by a partial restoration of the tetraploid hepatocyte nuclear population, this being rather more evident in the aflatoxin animals than in the 2-Me-DAB animals. Also, a peak with octaploid nuclei was observed later in the feeding regime in the case of rats fed the aflatoxin

FIG. 1.—Zonal nuclear profiles obtained during 6 weeks' feeding with toxic diet. (a–f) Aflatoxin $B_1$-containing diet for (a) 1 week (b) 2 weeks (c) 3 weeks (d) 4 weeks of feeding (e) 5 weeks (f) 6 weeks. (g–l) 2-Me-DAB containing diet for (g) 1 week (h) 2 weeks (i) 3 weeks (j) 4 weeks (k) 5 weeks (l) 6 weeks. Reverse diet: (m) 3 weeks of 2-Me-DAB followed by 3 weeks of aflatoxin $B_1$ (n) 3 weeks of aflatoxin $B_1$ followed by 3 weeks of 2-Me-DAB.

Light scattering at 254 nm is redrawn from original traces. Earlier part of profile (including membrane fractions) omitted. Average DNA content of nuclei contained in eluted peaks: Peak A = 8 pg DNA/nucleus (referred to as diploid peak in text); Peak B = 17 pg DNA/nucleus (referred to as tetraploid peak in text); Peak C = 32 pg DNA/nucleus (referred to as octaploid peak in text). For experimental details see Materials & Methods. Control profiles as in (a) and (g).

diet. In no case were changes in the ploidy distributions noted when the age-matched control groups on the non-toxic diet were used (Fig. 1(a) and (g)). It was observed in these studies that during the first 3 weeks of feeding with 2-Me-DAB the hepatic nuclear pellet isolated was stained orange. This staining was not evident in nuclei after 4 weeks of feeding with the 2-Me-DAB, and did not reappear during the remainder of the 6 weeks' feeding period. Fig. 1 also shows that, when 3 weeks' feeding with 2-Me-DAB was followed by 3 weeks' feeding with aflatoxin $B_1$, a zonal nuclear profile similar to 6 weeks' feeding with aflatoxin $B_1$ resulted. In the reverse schedule, in which the 2-Me-DAB feeding followed the aflatoxin, a nuclear zonal profile similar to 6 weeks' feeding with 2-Me-DAB resulted. The similarity in the appearance of the nuclear fraction obtained after 6 weeks' feeding with aflatoxin $B_1$ to that obtained when 3 weeks' feeding with aflatoxin $B_1$ followed 3 weeks' feeding with 2-Me-DAB can be seen from Fig. 2. Enlarged nuclei are present in Figs. 2(a) and (d) but not in (b) or (c).

Tissues removed from the livers at the time of the zonal centrifugation studies were subsequently examined histologically, the results being given in Table I. Although varying in severity, structurally similar lesions were observed in the tissues obtained from animals fed each of the two carcinogens, and they appeared at the same time in the feeding schedules. The most notable exception to this similarity was that aflatoxin $B_1$ resulted in a pronounced irregularity of size of hepatocyte nuclei, some being very large and bizarre, whereas this lesion was not obvious in sections of livers of animals fed 2-Me-DAB. Owing to limited animal-house facilities for feeding carcinogenic substances, it was not possible to retain more than a few animals from the groups used in the zonal centrifugation studies for examination of the subsequent incidence of hepatocarcinoma. However, in a subsequent experiment, animals of the same age as those used in the zonal experiment at the time of receiving the toxic diet, have been examined for tumour incidence 9 months after returning to normal diet. The results are given in Table II.

### DISCUSSION

The results given in Table II demonstrate that the requirement for a feeding

TABLE I.—*Histology of Liver Examined at Weekly Intervals during Feeding with Aflatoxin $B_1$ or 2-Me-DAB*

| Week | *Aflatoxin $B_1$* | *2-Me-DAB* |
| --- | --- | --- |
| 1 | Normal | Normal |
| 2 | Slight oval-cell proliferation | Slight bile-duct proliferation |
| 3 | Periportal necrosis. Extensive proliferation of oval cells and bile ducts. Variation in nuclear size. | Diffuse oval cell and bile duct proliferation. Parenchymal cell necrosis. Distorted lobular architecture. |
| 4 | Rapid proliferation of both oval cells and parenchymal cells, evidenced by frequent mitotic figures. Very extensive parenchymal-cell necrosis. No nodules. | Periportal bile-duct proliferation. Parenchymal-cell necrosis. Parenchymal-cell mitosis. No nodules. |
| 5 | Main feature repair of lesion. Parenchymal cells increased in number. Many large bizarre nuclei present. Hyperplastic nodules present. | Very extensive bile-duct proliferation. Parenchymal-cell necrosis. Hyperplastic nodules present. |
| 6 | Lesion has regressed. Increased proliferation of parenchymal cells and large nodules present. | Similar to 5 weeks. Very large hyperplastic nodules present. Parenchymal cell compression. |
| | *3 weeks' aflatoxin $B_1$ followed by 3 weeks' Me-DAB* | *3 weeks' Me-DAB followed by 3 weeks' aflatoxin $B_1$* |
| | Bile-duct proliferation. Hyperplastic nodules present. Large bizarre nuclei. | As in reverse schedule but lesions less marked. |

TABLE II.—*Incidence of Hepatic Neoplasms after Feeding with 2-Me-DAB or Aflatoxin $B_1$*

| Toxin | Duration of feeding (weeks) | Number of animals | Number with Hepatic Neoplasms |
| --- | --- | --- | --- |
| 2-Me-DAB | 3 | 9 | 0 |
| | 6 | 10 | 10 |
| Aflatoxin $B_1$ | 3 | 12 | 0 |
| | 6 | 12 | 9 |

period longer than 3 weeks with low levels of an azo dye or aflatoxin $B_1$ in order to induce hepatic neoplasm, reported by Hughes (1970) and Butler (1970) for Wistar rats, is also true for Fischer rats. Furthermore, the results of this study clearly demonstrate that, judged either by histological examination or by zonal centrifugation, the time course and nature of the changes in the livers of male Fischer rats induced by feeding low levels of aflatoxin $B_1$ or 2-Me-DAB appear similar. We have previously suggested, in the case of feeding aflatoxin $B_1$, that the first 3 weeks' sub-carcinogenic feeding period is necessary because, as a result of the accompanying prolonged inhibition of certain biochemical processes, principally nucleic acid synthesis, a large proportion of the hepatocyte population cannot survive longer than this period (Judah *et al.*, 1977). The length of this period could be dictated, for example, by the half-life of some essential species of m-RNA. On the present evidence, it is possible that similar considerations could apply to feeding 2-Me-DAB.

The effect of 3 weeks' feeding with the toxic diets is typified in the case of both carcinogens by bile-duct proliferation and parenchymal-cell necrosis. The zonal centrifugation profiles indicate that it is the hepatocytes containing tetraploid nuclei which account for a large percentage of the necrosing tissue. From the results given in Table II it is evident that these changes do not result in hepatocarcinoma. By 4 weeks, bile-duct proliferation is still proceeding, but parenchymal-cell proliferation has commenced, which at the 5 weeks' stage results in hyperplastic nodules. The zonal centrifugation profiles

FIG. 2.—Hepatic nuclear samples obtained after feeding toxic diets, and before placing on zonal rotor. (a) 6 weeks of aflatoxin $B_1$; (b) 6 weeks of 2-Me-DAB; (c) 3 weeks of aflatoxin $B_1$ followed by 3 weeks of 2-MeDAB; (d) 3 weeks of 2-Me-DAB followed by 3 weeks of aflatoxin $B_1$. Phase contrast ×370.

indicate that the proliferation of hepatocytes must result predominantly in diploid cells. It is evident that, if parenchymal cells are proliferating at 4 weeks and forming hyperplastic nodules at 5 weeks, then this proliferation is in the presence of a continuing supply of the carcinogen, which at 3 weeks was sufficient to kill a large proportion of the tetraploid hepatocytes. We have presented evidence, based on the results of cell-culture experiments, that the parenchymal cells which proliferate to take the place of those killed by treatment with aflatoxin $B_1$ are resistant to the cytotoxic action of this carcinogen (Judah *et al.*, 1977). Clearly, a similar situation could obtain in the case of the cells proliferating in the presence of continued feeding with 2-Me-DAB. The resistance to the cytotoxicity could clearly be of importance in the carcinogenic process in permitting cells to divide in the presence of the carcinogen, lesions in the DNA thus becoming permanently fixed.

The changes observed at 3–4 weeks of feeding 2-Me-DAB, in the colour of the nuclear pellet, suggest that, amongst other possibilities, the necrosis which takes place at this stage is of susceptible hepatocytes, whose nuclei contain bound dye, and their subsequent replacement is by a hepatocyte population whose nuclei exhibit little if any dye-binding. It has previously been reported that during amino-azo dye carcinogenesis a metabolite of the dye becomes bound to liver protein and the degree of this binding rises but subsequently falls (Miller and Miller, 1947) and the time of the fall in dye-binding corresponds to an increase in the mitotic index in the liver (Hughes, 1970).

On the present evidence of the zonal centrifugation studies and histological examinations, using animals fed one carcinogen for 3 weeks followed by 3 weeks' feeding with the other, it appears that the induction of resistance to the cytotoxicity of one of the carcinogens permits the development of hyperplastic growth in the presence of the other. In other words, the resistance to cytotoxicity is not confined to the original inducing agent. This could clearly have general relevance to chemical hepatocarcinogenesis. The effect of these reversed feeding schedules on the induction of hepatocarcinoma would clearly repay detailed investigation.

The authors wish to thank Mr D. J. Judah for carrying out the zonal centrifugations and Mr S. J. Gray and his staff for the histological preparations.

### REFERENCES

BUTLER, W. H. (1970) Liver Injury Induced by Aflatoxin. In *Progress in Liver Disease*, Vol. III, Eds. H. Popper and F. Schaffner. New York: Grune and Stratton, p. 141.

BUTLER, W. H. (1976) Early Cell Changes in Chemical Carcinogenesis. In *Fundamentals in Cancer Prevention*. Eds. P. N. Magee, S. Takayama, T. Sugimura and T. Matsushina. University of Tokyo Press, p. 89.

FARBER, E. (1973) Carcinogenesis, Cellular Evolution as a Unifying Thread. *Cancer Res.*, **33**, 2537.

GODOY, H. M., JUDAH, D. J., ARORA, H. L., NEAL, G. E. & JONES, G. (1976) The Effect of Prolonged Feeding with Aflatoxin $B_1$ on Adult Rat Liver. *Cancer Res.*, **36**, 2399.

HUGHES, P. E. (1970) Liver-cell Responses to the Carcinogen 3′methyl-4-dimethylaminoazobenzene. *Chem-Biol. Interactions*, **1**, 301.

JUDAH, D. J., LEGG, R. F. & NEAL, G. E. (1977) Development of Resistance to Cytotoxicity during Aflatoxin Carcinogenesis. *Nature, Lond.*, **265**, 343.

MILLER, E. C. & MILLER, J. A. (1947) The Presence and Significance of Bound Aminoazo Dyes in the Livers of Rats Fed p-Dimethylaminoazobenzene. *Cancer Res.*, **7**, 468.

NEAL, G. E., GODOY, H. M., JUDAH, D. J. & BUTLER, W. H. (1976) Some Effects of Acute and Chronic Dosing with Aflatoxin $B_1$ on Rat-liver Nuclei. *Cancer Res.*, **36**, 1771.

WIDNELL, C. C. & TATA, J. R. (1964) A Procedure for the Isolation of Enzymically Active Rat Liver Nuclei. *Biochem. J.*, **92**, 313.